# Smad3 Mediates Renal Fibrosis via GPX4-Dependent Ferroptosis

**DOI:** 10.7150/ijbs.114075

**Published:** 2025-09-22

**Authors:** Kaixiang Liu, Min Yu, Yangyang He, Yi Li, Xiao-ru Huang, Guisen Li, Li Wang, Hui-yao Lan, Xiang Zhong

**Affiliations:** 1 Department of Nephrology, Sichuan Provincial People's Hospital, School of Medicine, University of Electronic Science and Technology of China, Chengdu, China.; 2 Departments of Medicine & Therapeutics, The Chinese University of Hong Kong, Hong Kong, and Guangdong-Hong Kong Joint Laboratory for Immunological and Genetic Kidney Disease, Guangdong Academy of Medical Science, Guangdong Provincial People's Hospital, Guangzhou, China.

**Keywords:** GPX4, Smad3, Renal fibrosis, Ferroptosis, TGF-β1.

## Abstract

TGF-β/Smad3 signaling is a key pathway leading to the cell death and renal fibrosis. Here we report a new mechanism through which Smad3 mediates renal fibrosis by downregulating the glutathione peroxidase 4 (GPX4), a central inhibitor for ferroptosis. In patients with chronic kidney disease (CKD) and a mouse model of unilateral ureteral obstruction (UUO), progressive renal fibrosis was associated with the overactive Smad3 signaling and the development of ferroptosis identified by decreased GPX4 while increasing two ferroptosis biomarkers including the Transferrin receptor 1 (TFR1) and 4-Hydroxynonenal (4-HNE). Mechanistically, we uncovered that Smad3 could bind directly to GPX4 to repress its transcription while increasing TFR1 and 4-HNE expression, which was abolished when this binding site was mutated. This novel finding was functionally confirmed in the UUO mice and mouse embryonic fibroblasts (MEFs) in which deletion of Smad3 protected against UUO and transforming growth factor-β1 (TGF-β1)-induced loss of GPX4, upregulation of TFR1 and 4-HNE, and progressive renal fibrosis *in vivo* and *in vitro*. Importantly, we also found that GPX4 was a downstream target gene of Smad3 and functioned to protect against Smad3-mediated renal fibrosis as silencing GPX4 restored UUO-induced severe renal fibrosis in Smad3 KO mice and in TGF-β1-stimulated Smad3 KO MEFs and SIS3-treated HK-2 cells. Thus, GPX4 is protective in renal fibrosis. Smad3 mediates renal fibrosis via a mechanism associated with GPX4-dependent ferroptosis. The protective effect of GPX4 on Smad3-mediated renal pathologies suggests that targeting the Smad3/GPX4 axis may be a novel therapy for CKD.

## Introduction

Renal fibrosis is a major pathological feature in chronic kidney disease (CKD) and is characterized by excessive accumulation of extracellular matrix (ECM) components in the kidney.^1^ In clinical practice, renal fibrosis is an important and reliable indicator for the progression of CKD patients.^2^ Therefore, it is critical to alleviate renal fibrosis to prevent or slow the progression of CKD to end-stage renal disease (ESRD).

Renal fibrosis is a complex and multifactorial process involving various cellular and molecular mechanisms. 3,4 Renal fibrosis is associated with the activation of fibroblasts, which differentiate into myofibroblasts, causing changes in cell morphology and increased production of fibrosis markers such as alpha-smooth muscle actin (α-SMA) and collagen. This process is regulated by several signaling pathways, including the transforming growth factor beta (TGF-β)/Smad3 signaling pathway. 3-5 TGF-β1 can also promote the differentiation of fibroblasts and macrophages into myofibroblasts, worsening renal fibrosis. 4, 5

Smad3, a key downstream mediator of TGF-β1, is highly activated in fibrotic tissues including the kidney and exerts its biological activities through Smad3-binding elements, which are found in most collagen promoters 6. Smad3 can be activated by TGF-β1 and other profibrotic cytokines and mediators including angiotensin II, advanced glycation end products (AGE), and c-reactive protein (CRP) via TGF-β-dependent and independent signaling pathways including the ERK/p38 MAPK and NF-κB pathways, to mediate renal inflammation and fibrosis.^5^,^7^-^10^ The findings that genetic deletion of Smad3 protects against the development of obstructive and diabetic nephropathy reveal a functional role of Smad3 in renal fibrosis.^11^-^13^ Mechanistically, Smad3 mediates renal fibrosis by regulating the downstream miRNAs/lncRNAs.^14^-^17^ Recent studies also detected that Smad3 can induce acute kidney injury (AKI) via a NLRP3-dependent mechanism and causes the cell death via apoptosis, necroptosis, and autophagy in mice with AKI and CKD by activating the p21/p27 and Ripk3/MLKL pathways or by triggering lysosome depletion.[8, 18-21]Thus, targeting Smad3 can effectively protect against AKI and CKD via mechanisms associated with inhibiting the cell death and fibrosis pathways. 18-25

Glutathione peroxidase 4 (GPX4) is an antioxidant enzyme and a central inhibitor of ferroptosis, a recently discovered non-apoptotic form of cell death induced by iron-dependent lipid peroxidation.^26^ Transferrin receptor 1 (TFR1), a key receptor for iron uptake, is a critical biomarker of ferroptosis and functions to promote the Fenton reaction and lipid peroxidation 27. Ferroptosis is also related to lipid peroxidation and the 4-Hydroxynonenal (4-HNE) is a terminal product of lipid peroxidation and is also often used as a biomarker for ferroptosis 28-30. It has been demonstrated that loss of GPX4 contributes to the development of renal fibrosis with extensive ferroptosis, suggesting a protective role for GPX4 in the development of CKD.^31^-^34^ In addition, a recent study also demonstrated that inducible disruption of GPX4 causes acute renal failure with extensive tubular cell apoptosis and ferroptosis.^35^ Furthermore, specifically silencing renal GPX4 can abolish the protective effect of nuciferine on ferroptosis in folic acid-induced acute kidney injury in mice.^36^ Thus, targeting ferroptosis may be a new therapeutic strategy for progressive renal fibrosis.^37^-^40^ However, role of GPX4 in CKD, particularly ferroptosis-associated renal fibrosis, remains largely unclear and the molecular mechanisms that regulate expression of GPX4 during renal fibrosis remain unexplored.

Thus, in this study, we aimed to uncover the mechanistic link between TGF-β/Smad3 signaling and GPX4-dependent ferroptosis in the development of renal fibrosis. We first examined the relationship between GPX4 expression and activation of TGF-β/Smad3 signaling in the fibrotic kidney of patients and mice with CKD. Through comprehensive approaches using Smad3-knockout (Smad3-KO) mouse model of UUO and Smad3-KO mouse embryonic fibroblasts (MEFs), we then investigated the regulatory mechanism of TGF-β/Smad3 signaling in GPX4 expression during the development of renal fibrosis *in vivo* and *in vitro*. Finally, we explored the potential role of GPX4 in Smad3-driving renal fibrosis by specifically silencing GPX4 in the UUO kidney of Smad3-KO mice and Smad3-KO mouse embryo fibroblasts (MEFs).

## Materials and Methods

### Patients

Fifty-six patients diagnosed with CKD and biopsy-proven renal fibrosis between 2016 and 2021, including 20 patients with focal proliferative IgA nephropathy (IgAN), 19 patients with diabetic kidney disease (DKD), and 17 patients with minimal change disease (MCD) without renal fibrosis, were enrolled in this study. CKD was defined as abnormalities of kidney histology and function over the period for ≥3 months following the Kidney Disease: Improving Global Outcomes (KDIGO) 2012 guidelines (http://kdigo.org/home/guidelines/). Renal biopsies were independently evaluated by two senior pathologists at the Sichuan Provincial People's Hospital. The included patients had no history of receiving immunosuppressive drugs or corticosteroids when they underwent biopsy and had no other systemic diseases, such as rheumatoid arthritis, pulmonary fibrosis, or liver cirrhosis. This study was conducted in compliance with the Declaration of Helsinki and approved by the Medical Ethics Committee of the Sichuan Academy of Medical Sciences and Sichuan Provincial People's Hospital (No. 28 2019 Natural Science). Informed consent was obtained from all patients.

### Bioinformatics analysis by RNA-sequencing

Gene expression omnibus (GEO) datasets were obtained from the GEO database (https://www.ncbi.nlm.nih.gov/geo/). Differentially expressed RNAs were determined with the Limma package in R software. Differentially expressed genes were defined as genes with a p adj-value <0.05 and logFC ≥1. Functional categories, the association between genes, the corresponding Gene Ontology (GO) classification, and Kyoto Encyclopedia of Genes and Genomes (KEGG) enrichment analysis were analyzed using R software (version 4.3.1). Genes associated with ferroptosis were identified from the FerrDB database (http://www.zhounan.org/ferrdb/legacy/). Graphs were plotted using an online platform for data analysis and visualization (https://https://www.xiantaozi.com). Clean reads were aligned to the full-length sequenced transcriptome and reference genome transcripts using Minimap2. Fragments per kilobase (kb) of transcript sequence per million mapped reads were used as a measure of transcripts or gene expression. Differential expressions among sample groups were analyzed with DESeq2. Selected genes with a *p*-value <0.05 were considered significant.

### A mouse model of UUO induced in Smad3 WT and KO mice

Smad3-wild-type (Smad3-WT) and Smad3-KO mice with a C57BL/6 J background (male, aged 8-12 weeks) (n=6 each group) were used in this study. Smad3-KO mice (exon 8 deleted and exon 7 disrupted) were kindly provided by Dr. Chuxia Deng.^41^ To study the regulatory role of Smad3 in the expression of GPX4 during renal fibrosis, the mice were randomly allocated into the Sham group and UUO group. UUO surgery was performed using left ureteral ligation to establish an *in vivo* renal fibrosis model (n=6), as previously described.^42^ Sham mice (n=6), as the control group, underwent the same procedure as the UUO mice, without ligation of the left ureter.

All experimental animal procedures were approved by the Animal Experimentation Ethics Committee of the Chinese University of Hong Kong (CUHK) (AEEC Ref No.: 22-068-MIS) and were carried out in accordance with the Guide for the Care and Use of Laboratory Animals. All mice were housed by the Laboratory Animal Service Center of CUHK in an environment maintained at 22-23 °C with <70% relative humidity and a 12 h light/dark cycle with free access to standard mouse diet and water.

### Ultrasound-microbubble-mediated inhibition of renal GPX4

A well-established method of ultrasound-microbubble-mediated gene transfer was used to effectively transfect the GPX4 shRNA plasmids into the kidney. The detailed methodology and transfection efficiency have been previously described.^14^, ^16^-^19^, ^23^, ^43^, ^44^ In this study, the GPX4 shRNA plasmid at 200 μg/mouse or empty vector control in 200 μL saline was mixed with lipid microbubbles (SonoVue; Bracco, Milan, Italy) at a ratio of 1:1. A 400 μL aliquot of the mixture was injected into the mice via the tail vein, followed immediately by placing the ultrasound probe (Therasonic 450; Electro Medical Supplies, Wantage, United Kingdom) on their backs, opposite their bilateral kidneys with a pulse-wave output of 1 MHz at 2 W/cm^2^ for a total of 10 min by rotating to each side in a 30-s interval, as previously described. 14, 16-19, 23, 43, 44 Mice were sacrificed on day 7 after UUO surgery for determining GPX4, ferroptosis markers, and fibrosis.

### Cell culture

Human tubular epithelial (HK-2) cells were purchased from the American Type Culture Collection (Manassas, VA, USA). Smad3 WT/KO MEFs were kindly provided by Dr EP Bottinger and Dr E Piek (NIH) and have been well characterized previously.^6^ Cells were cultured in Dulbecco's modified Eagle's medium (DMEM) (Gibco, Grand Island, NY, USA) supplemented with 10% FBS, 100 U/mL penicillin, and 100 µg/mL streptomycin sulfate. To study the regulatory mechanisms of Smad3 in GPX4-induced ferroptosis, Smad3 WT/KO MEFs with or without GPX4 shRNA plasmid transfection were cultured in DMEM with or without TGF-β1 (5 ng/mL, Peprotech 100-21). HK-2 cells with or without GPX4 shRNA plasmid transfection were cultured in DMEM/F12 medium (Gibco) with or without TGF-β1(5 ng/mL) in the presence or absence of a Smad3 inhibitor SIS3 (5 µm, Selleck, S7959), which was added into the cells at 1 hour before stimulation with TGF-β1.

### Immunohistochemistry

Immunohistochemistry was performed on paraffin-embedded kidney sections (3 µm) using a microwave-based antigen retrieval technique.^45^ Sections were incubated with primary antibodies, including anti-p-Smad3 (600-401-919; Rockland), anti-GPX4 (ab125066, Abcam), anti-4-HNE (BS-6313R, Bioss), anti-TFR1 (ab214039, Abcam), anti-α-SMA (19245S, Cell Signaling Technology), anti-collagen-1 (Col-1) (1310-01, SouthernBiotech), and anti-fibronectin (FN) (ab2413, Abcam) antibodies, at 4 °C overnight. Sections were washed and incubated with donkey anti-goat IgG H&L (HRP polymer) (ab214881, Abcam) or EnVision+ System-HRP labeled polymer anti-rabbit (K4003, DAKO) for 1 h at room temperature. Color was developed with 3,3'-diaminobenzidine (DAB) (045-22833, FujiFilm Wako Pure Chemical Corporation). Sections were viewed under a Leica CRT6000 Light Microscope.

### Chromatin immunoprecipitation (ChIP)

HK-2 cells were stimulated with 5 ng/mL TGF-β1. ChIP was performed using the SimpleChIP® Plus Enzymatic Chromatin IP Kit (CST, USA), according to the manufacturer's instructions. Smad3 antibody (9523, Cell Signaling Technology) was used for immunoprecipitation. The GPX4 promoter primer was forward: 5′- GCCAAGATGTGGGGCAGTA-3′, reverse: 5′- CGCGGTATGTGCTCAGAAA-3′.

### Dual-luciferase reporter assay

The human GPX4 promoter region (-720 to -7 bp) was cloned into the pGL3-basic vector to generate pGL3-GPX4. Pro. A point mutation was then introduced into the Smad3 binding site within this promoter to create pGL3-GPX4.Pro.mut. For the dual-luciferase reporter assay, HEK293T cells were seeded onto the 24-well plate at a density of 7.5 x 10^4^ cells per well. Cells were transfected with 500 ng pGL3-Basic or pGL3-GPX4.pro or pGL3-GPX4.pro.mut, along with pcDNA3.1-Smad3 to overexpress human Smad3. To normalize the transfection efficiency, 50 ng pGMLR-TK *Renilla* luciferase reporter plasmid was included in each transfection. Six hours post-transfection, the medium was replaced, and cells were incubated for additional 48 hours. The cells were subsequently lysed, and luciferase activity was measured using the TransDetect® Double-Luciferase Reporter Assay Kit (FR201-01, TransGen Biotech) and quantified in a luminometer (EnVision XCite 2105, PerkinElmer). The transcriptional activity of the cloned GPX4 promoter was calculated as the ratio of firefly luciferase to *Renilla* luciferase. Each treatment was performed in triplicate.

### Western blot analysis

Total proteins from mouse kidney tissues and MEFs were extracted using RIPA lysis buffer (P0013B; Beyotime). Proteins were electrophoresed on a 10-15% SDS-PAGE gel and transferred to a nitrocellulose transfer membrane (66485; Pall Corporation). After blocking, membranes were incubated with primary antibodies at 4°C overnight, including rabbit GPX4 monoclonal antibody (ab125066; Abcam), anti-Smad3 (51-1500; Invitrogen), anti-p-Smad3 (ab52903; Abcam), anti-4-HNE (BS-6313R, Bioss), anti-TFR1 (ab214039, Abcam), anti-Col-1 (BS-10423R, Bioss) and anti-FN (ab2413, Abcam) antibodies, mouse α-SMA monoclonal antibody (19245S, Cell Signaling Technology), and anti-β-actin antibody (SC-69879; Santa Cruz). Membranes were incubated with anti-rabbit IgG (H&L) conjugated to DyLight 800 (611-145-002; Rockland) or anti-mouse IgG (H&L) conjugated to DyLight 800 (610-145-002; Rockland). Protein expression levels were visualized using the Odyssey Infrared Imaging System (San Diego, CA, USA) and quantitatively analyzed using ImageJ software (National Institutes of Health, Bethesda, MD, USA).

### Real-time polymerase chain reaction (PCR)

Total RNA from mice kidneys and HK-2 cells was extracted using TRIzol reagent (TR118; Molecular Research Center), according to the manufacturer's instructions. Real-time quantitative PCR was performed using QuantStudio 6 and 7 Flex Real-time PCR systems (4489826; Thermo Fisher Scientific) and SYBR Green Supermix (1725122; Bio-Rad). Primers were: mouse GPX4 forward: 5′-CGCCCGAGGTACGCAATAG-3′, reverse: 5′-GCAGTTGGGTTGGAAGGACT-3′; mouse β-actin: forward 5′-GTGACGTTGACA T CCGTAAAGA-3′, reverse 5′-GCCGGACTCATCGTACTCC-3′. Other primers were as previously described. 17, 46, 47 β-actin was the internal control.

### Statistical analysis

All values from this study are expressed as mean ± SD. Statistical analyses were performed by one-way ANOVA using GraphPad Prism9 (GraphPad Software, San Diego, CA, USA). Means among multiple groups were compared using the Benjamini-Hochberg method. Correlation analysis was analyzed using Pearson's correlation test. Means among the two groups were compared with the t-test. Statistical significance was set at *P* < 0.05.

## Results

### Smad3 activation is associated with reduced expression of GPX4 while increasing ferroptosis and progressive renal fibrosis in CKD patients and mouse model of UUO

**Figure 1:**
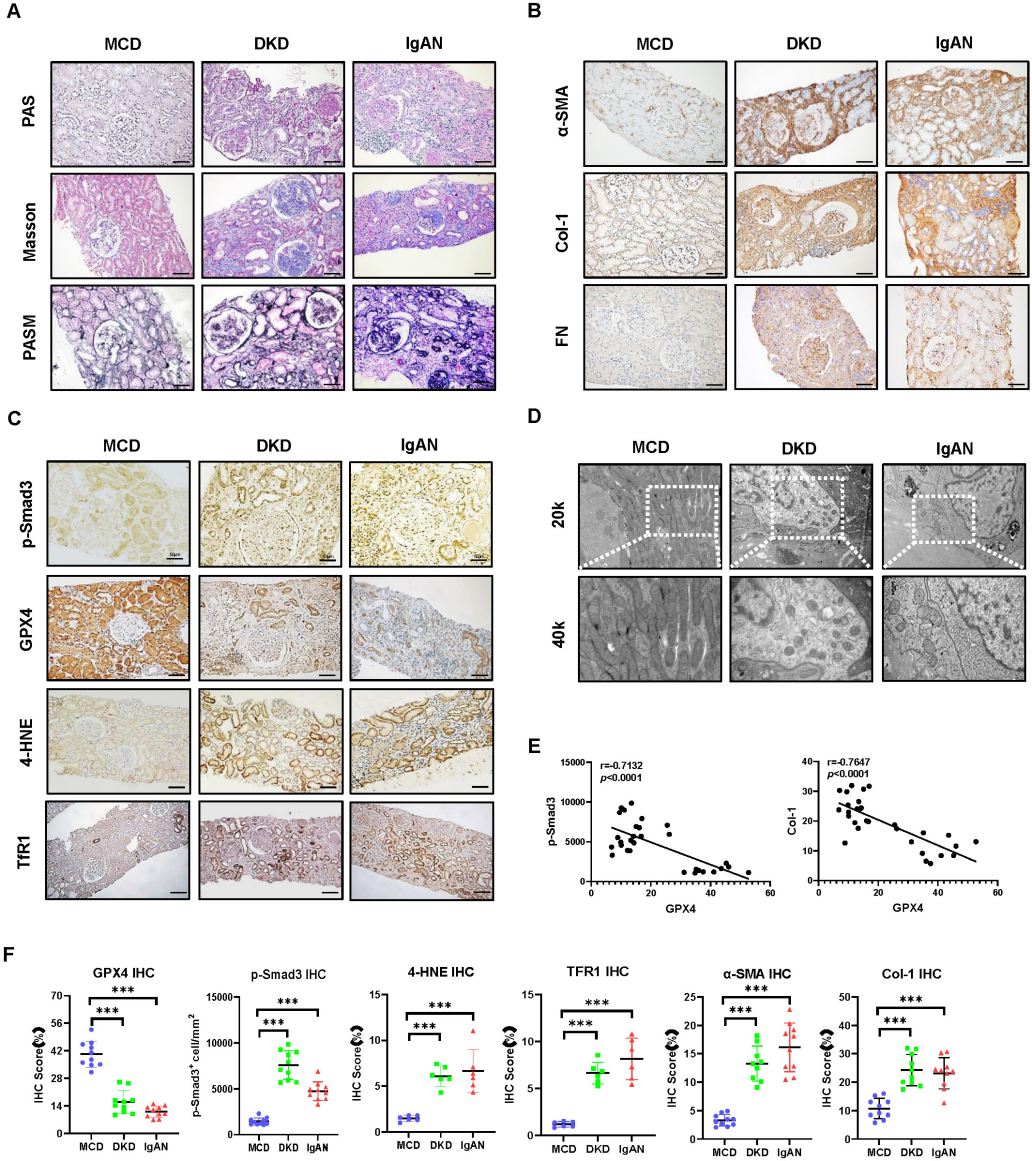
** Smad3 signaling is highly activated in CKD patients with progressive renal fibrosis and is associated with decreased GPX4 while increasing ferroptosis. (A)** Representative photomicrographs of kidney sections (periodic acid-Schiff, Masson's trichrome, and periodic acid-silver methenamine staining) from patients with MCD, IgAN, and DKD. **(B)** Representative photomicrographs of immunohistochemistry staining of renal α-SMA, Col-1, and FN. **(C)** Representative photomicrographs of immunohistochemistry staining of renal p-Smad3, GPX4, 4-HNE and TFR1. **(D)** Representative transmission electron microscope (EM) photomicrographs of pathological changes in the mitochondria. **(E)** Correlation of GPX4 with p-Smad3 and the fibrosis marker Col-1 in the kidney of patients with CKD. **(F)** Quantitation of renal GPX4, p-Smad3, 4-HNE, TFR1, α-SMA, and Col-1 protein expression. Each dot presents one patient, and data were represented as mean ± SD. ***P < 0.001. Scale bar, 100 μm.

To investigate whether alteration of GPX4 is associated with renal ferroptosis and fibrosis in patients with CKD, we collected kidney tissues from patients with CKD and biopsy-proven renal fibrosis, including patients with DKD and focal proliferative IgAN. Kidney tissue from patients with MCD without renal fibrosis was collected as a control. Routine pathological staining, including periodic acid-Schiff (PAS), Masson's trichrome, and periodic acid-silver methenamine (PASM), showed that kidney tissues from patients with DKD and IgAN exhibited glomerulosclerosis, renal interstitial fibrosis with abundant collagen matrix deposition (**Figure 1A**).

**Figure 2:**
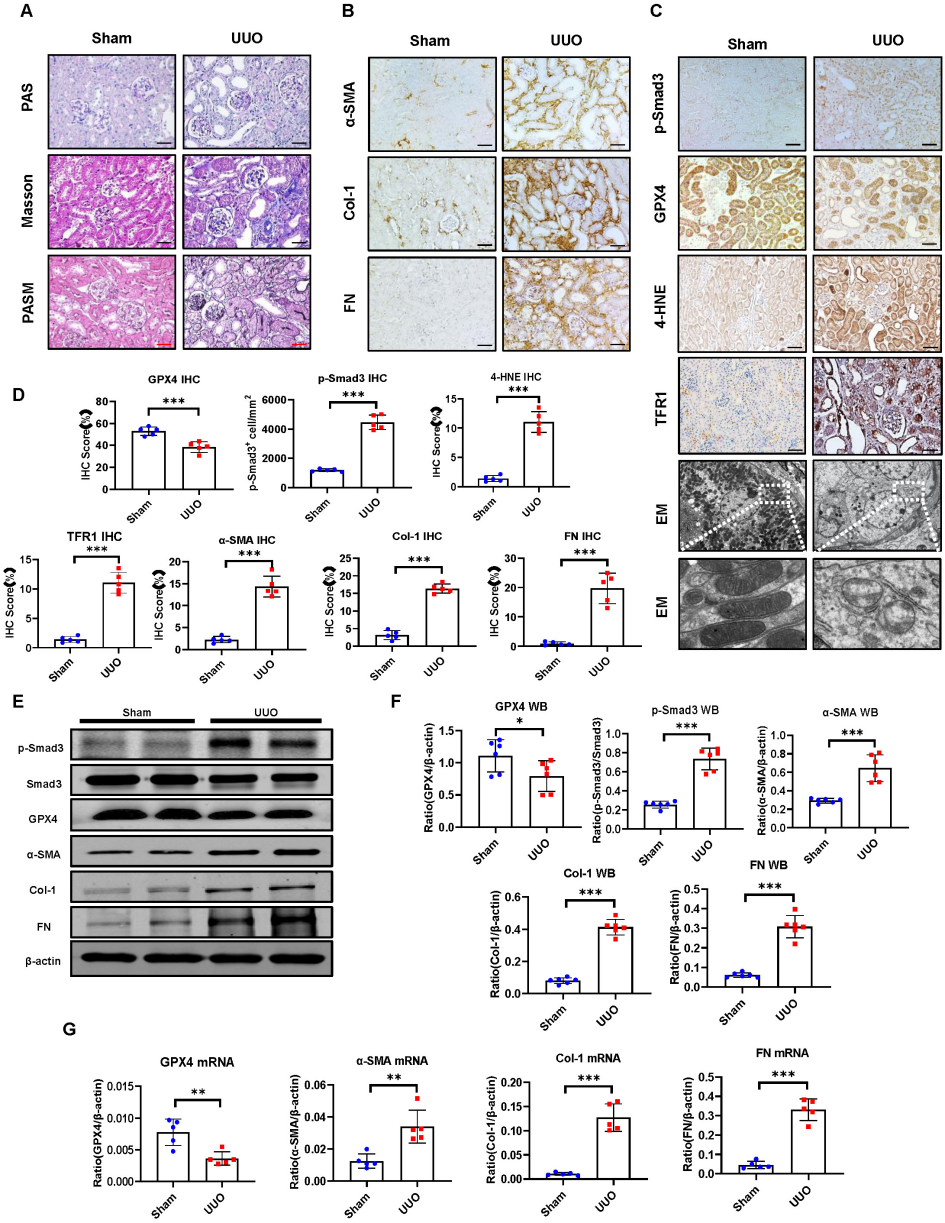
** Smad3 is highly activated in the mouse UUO kidney with progressive renal fibrosis, which is associated with decreased renal GPX4 while increasing ferroptosis. (A)** Representative photomicrographs of kidney sections (periodic acid-Schiff, Masson's trichrome, and periodic acid-silver methenamine staining) from Sham and UUO mice. **(B)** Representative photomicrographs of immunohistochemistry staining of renal α-SMA, Col-1, and FN. **(C)** Representative photomicrographs of immunohistochemistry staining of renal p-Smad3, GPX4, 4-HNE, TFR1, and representative transmission electron microscope photomicrographs. **(D)** Quantitation of renal p-Smad3, GPX4, 4-HNE, TFR1, α-SMA, Col-1, and FN protein expression. **(E)** Western blotting for p-Smad3, Smad3, GPX4, 4-HNE, TFR1, α-SMA, Col-1, and FN. **(F)** Quantitation of renal p-Smad3, GPX4, α-SMA, Col-1, and FN protein expression.** (G)** Real-time PCR for renal GPX4, α-SMA, Col-1, and FN mRNA expression. Data are presented as mean ± SD for groups of 6 mice. *P < 0.05, **P < 0.01, ***P < 0.001; Scale bar, 50 μm.

Patients in the control group showed minimal morphological changes without evidence of obvious renal fibrosis **(Figure 1A)**. Immunohistochemistry revealed that renal fibrosis, evidenced by increased α-SMA^+^ myofibroblasts and Col-1 and FN accumulation, was associated with substantial activation of p-Smad3, downregulation of GPX4, and increased biomarkers of ferroptosis as demonstrated by upregulation of 4-HNE and TFR1 in patients with DKD and IgAN (**Figure 1B, C, F**). Correlation analysis showed that GXP4 expression inversely correlated with activation of Smad3 signaling (p-Smad3) and the fibrosis marker Col-1 **(Figure 1E).** Electron microscopy (EM) found morphological alterations associated with ferroptosis, such as a reduction in mitochondria quantity, an increase in bilayer density, and decrease in mitochondrial cristae in the kidney tissues of patients with DKD and IgAN but not in patients with MCD (**Figure 1D**). These renal pathologies were also observed in a mouse model of UUO. Histology (PAS, Masson's trichrome, and PASM), immunohistochemistry, western blot, and real-time PCR detected that all UUO mice developed severe renal fibrosis with a marked upregulation and excessive accumulation of α-SMA, Col-1 and FN on day 7 (**Figure 2).** These fibrotic changes were associated with a marked activation of Smad3 and decreased renal GPX4 expression while largely increasing ferroptosis biomarkers such as 4-HNE and TFR1 (**Figure 2, Figure S1A and B).** Electron microscope also showed a reduction in mitochondria quantity, absent or decreased mitochondrial cristae, increased membrane density, and ruptured outer mitochondrial membranes **(Figure 2C).**

**Figure 3:**
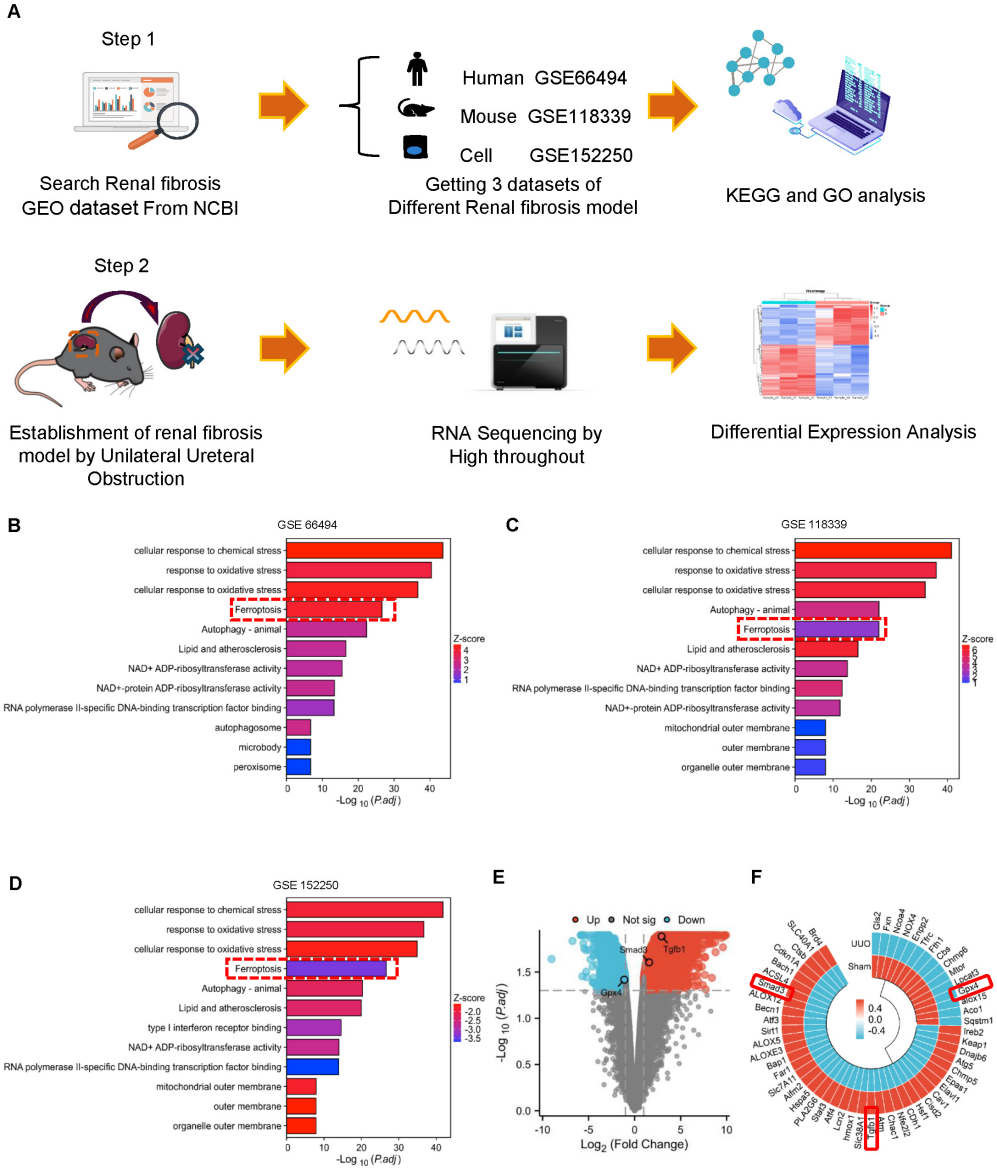
** Bioinformatics analysis reveals that decreased renal GPX4 expression and the development of ferroptosis are associated with TGF-β/Smad3 signaling in the fibrotic kidney of CKD patients and UUO mice. (A)** Three different renal fibrosis datasets (GSE66494, GSE118339, and GSE152250) were obtained from the NCBI for KEGG and GO analyses. C57BL/6 mice were used to establish a renal fibrosis model using UUO surgery. The kidneys were used for RNA sequencing (RNA-seq) 7 days later. **(B) - (D)** Over-representation analysis of GO Biological Processes and KEGG on significantly (adjusted *P*-value < 0.05) differentially expressed genes with upregulated (log2-fold change > 0.5) and downregulated (log2-fold change < -0.5) expression in kidney tissues from patients with CKD **(B)** or from UUO mice** (C),** and TGF-β1-treated tubular epithelial cells (TEC) **(D)**.** (E)** Volcano plot depicting differentially expressed genes (adjusted *P*-value < 0.05 and 1 log2-fold change cutoff) in kidney tissues between Sham and UUO mice.** (F)** Heatmap and hierarchical clustering of selected differentially expressed genes involved in ferroptosis and TGF-β/Smad3 signaling. Each experiment was performed in duplicate, and kidney samples were used from two Sham or UUO mice.

Thus, in CKD patients and mice, Smad3 activation is associated with decreased GPX4 expression, which may contribute to ferroptosis and progressive renal fibrosis. These findings suggest a close link between TGF-β/Smad3 signaling and GPX4-dependent-ferroptosis during the development of renal fibrosis. This was further examined by bioinformatics analysis with the Kyoto Encyclopedia of Genes and Genomes (KEGG) and Gene Ontology (GO) on three different renal fibrosis datasets from the National Center for Biotechnology Information (NCBI) and RNA-sequencing in a mouse model of UUO **(Figure 3A).** Results showed that ferroptosis is an important biological process in the development of renal fibrosis **(Figure 3B-D).** Next, we focused on genes involved in ferroptosis and found that GPX4 gene expression was significantly decreased in the UUO mouse kidney, which was associated with activation of the TGF-β/Smad3 pathway **(Figure 3E, F).** Results from the bioinformatics analysis again support the finding that there is close link between activation of TGF-β/Smad3 signaling and GPX4-dependen ferroptosis during the development of renal fibrosis.

### Smad3 interacts directly with GPX4 to suppress its expression, resulting in the development of ferroptosis-associated renal fibrosis in a mouse model of UUO and in TGF-β1-stimulated MEFs

**Figure 4:**
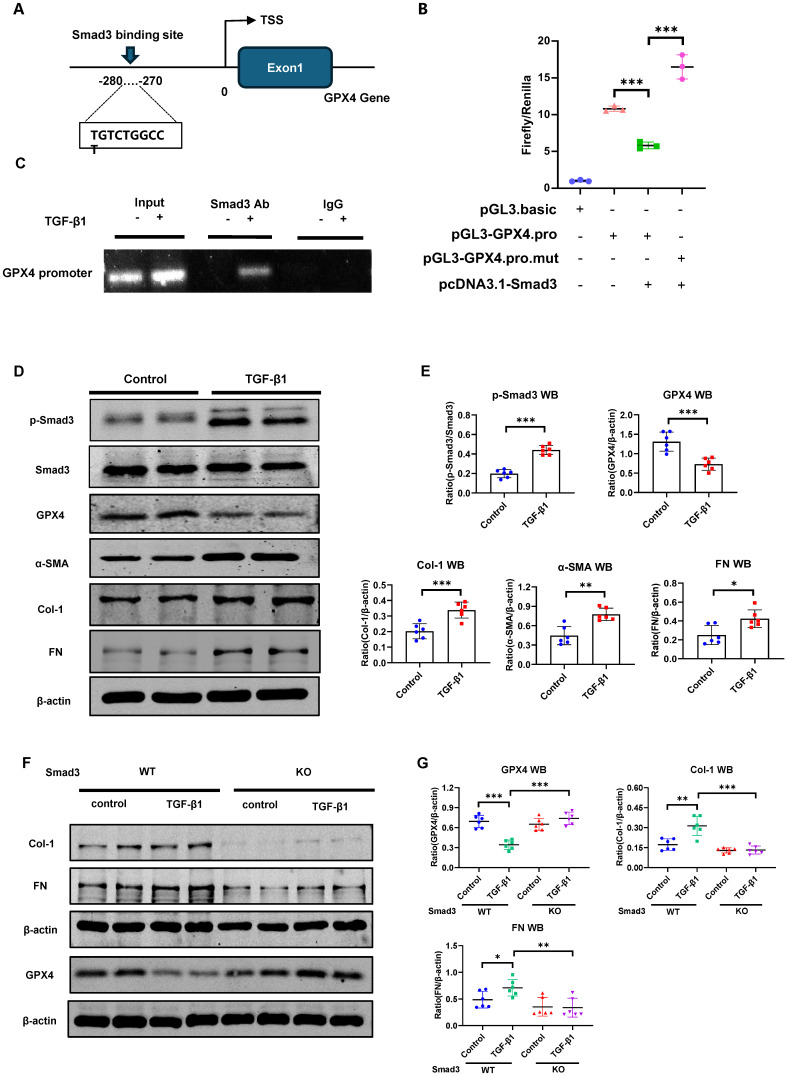
** GPX4 is a Smad3 target gene for fibrosis and is negatively regulated by TGF-β1 via a Smad3-dependent manner in HK-2 cells and MEFs**. **(A)** A Smad3 binding site was predicted on the promoter region of the GPX4 genomic sequence by the JASPAR database. **(B)** Dual-luciferase reporter assay shows the transcriptional regulation of Smad3 on GPX4 expression in HEK293T cells. Note that mutation of the Smad3 binding site protects against the transcriptional repression of GPX4. **(C)** The ChIP assay showed the physical binding of Smad3 on the GPX4 promoter genomic sequence, which is enriched after TGF-β1 treatment in HK-2 cells. **(D, E)** Western blot and quantitative analysis show that treatment with TGF-β1 (5 ng/mL) induces activation of Smad3 (p-Smad3) and upregulation of Col-1 and FN, which is associated with inhibition of GPX4 expression in MEFs. **(F, G)** Western blot analysis detects that that deletion of Smad3 enhances GPX4 expression but inhibits TGF-β1-induced fibrosis including Col-1, and FN in Smads3 KO MEFs. Data are presented as mean ± SD for at least 3 independent experiments. *P < 0.05, **P < 0.01, ***P < 0.001.

Next, we determine whether Smad3 can interact with GPX4 to regulate its expression. As expected, addition of TGF-β1 was capable of inducing p-Smad3 nuclear translocation (**Figure S2**), suggesting that Smad3 may transcriptionally regulate ferroptosis. This was further demonstrated by the finding of a Smad3 binding site identified on the promoter region of GPX4 gene using the JASPAR (https://jaspar.genereg.net/) (**Figure 4A).** ChIP assay further revealed that Smad3 could bind GPX4 directly, which was enhanced in response to TGF-β1 in HK-2 cells (**Figure 4C**). Dual-luciferase assay in HEK293T cells revealed that overexpression of Smad3 largely suppressed *GPX4* transcription, which was abrogated when the Smad3 binding site was mutated (**Figure 4B**), providing direct evidence for the suppressive role of Smad3 in *GPX4* transcription. This was further demonstrated in Smad3 KO MEFs and SIS3-treated HK-2 cell. Addition of TGF-β1 stimulation caused Smad3 phosphorylation (p-Smad3), which was associated with a marked decrease in GPX4 while upregulating 4-HNE, TFR1, α-SMA, Col-1 and FN in Smad3 WT MEFs (**Figure 4D, E** and **Figure S1C, D**) and in HK-2 cell (**Figure S3**). In contrast, genetic deletion or pharmacological inhibition of Smad3 abolished TGF-β1-induced loss of GPX4 and upregulation of 4-HNE, TFR1, α-SMA, Col-1, and FN as observed in Smad3 KO MEFs (**Figure 4 F-G, Figure S4A**) and in SIS3-treated HK-2 cells (**Figure S3**). These findings suggest that GPX4 is negatively regulated by TGF-β1 via a Smad3-dependent mechanism and Smad3 may directly interact to GPX4 to repress it expression during renal fibrosis.

**Figure 5:**
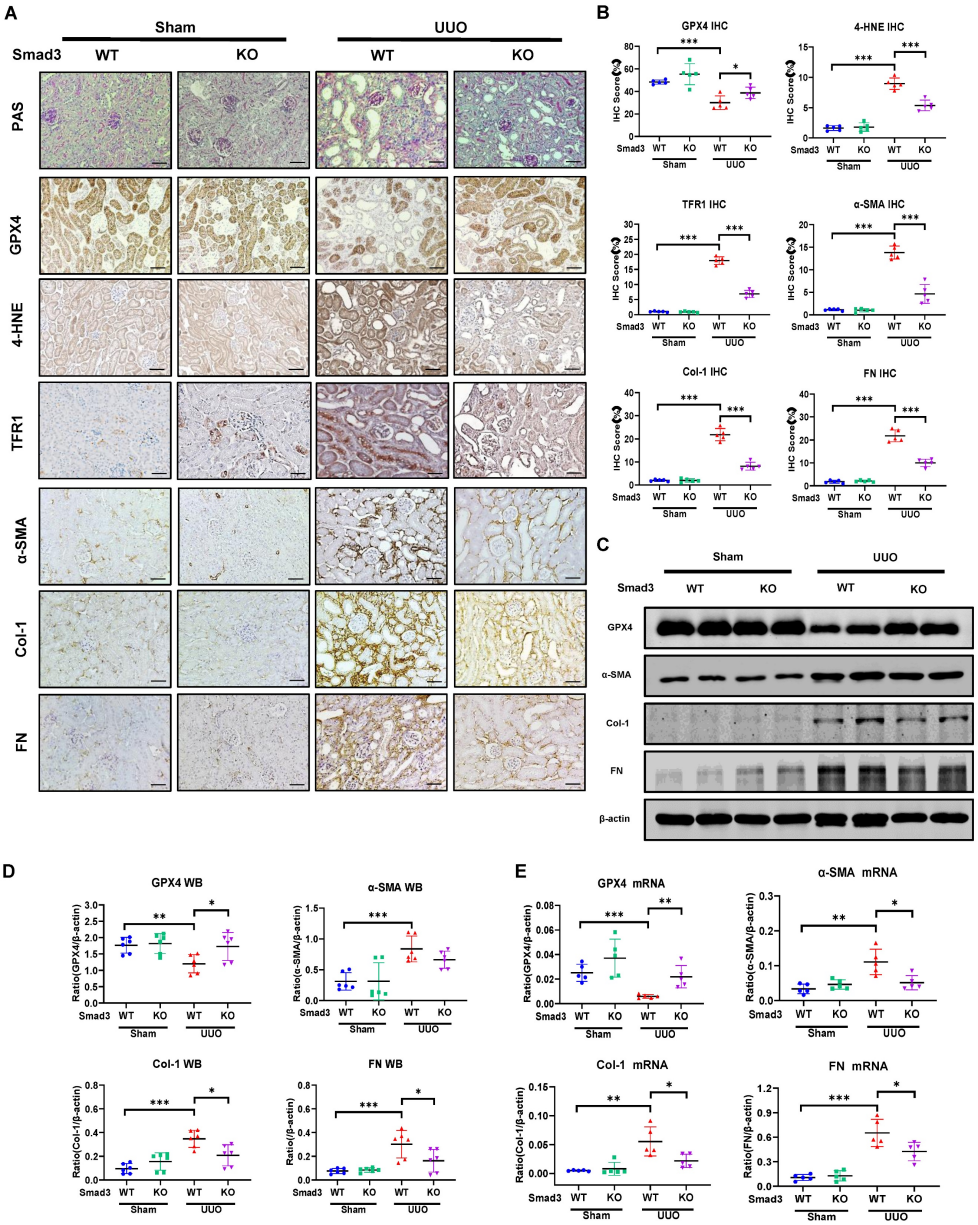
** Mice null for Smad3 are protected from UUO-induced loss of GPX4, thereby inhibiting ferroptosis and progressive renal fibrosis in a mouse model of UUO. (A)** Representative photomicrographs of kidney sections from Smad3 WT and Smad3 KO UUO mice on 7 days after surgery. Sections were stained with PAS and antibodies against GPX4, 4-HNE, TFR1, α-SMA, Col-1, and FN by immunohistochemistry. **(B)** Quantitation of renal GPX4, 4-HNE, TFR1, α-SMA, Col-1, and FN protein expression. **(C)** Western blotting for expression of GPX4, α-SMA, Col-1, and FN in the UUO kidney. **(D)** Quantitation of renal GPX4, α-SMA, Col-1, and FN protein expression. **(E)** Real-time PCR for renal GPX4, α-SMA, Col-1, and FN mRNA expression. Data are presented as mean ± SD for groups of 6 mice. *P < 0.05, **P < 0.01, ***P < 0.001. Scale bar, 50 μm.

We then investigated the regulatory mechanism of Smad3 in GPX4-dependent ferroptosis during renal fibrosis in a mouse model of UUO induced in Smad3 KO/WT mice. Consistent with the findings in Smad3 KO/WT MEFs and SIS3-treated HK-2 cell, disrupted Smad3 largely protected against the loss of renal GPX4 and therefore suppressed expression of 4-HNE and TFR1 and the development of severe renal fibrosis as demonstrated by excessive accumulation of α-SMA, Col-1 and FN in a mouse model of UUO (**Figure 5** and **Figure S4B**)**.** These findings imply that Smad3 may suppress GPX4, a central inhibitor of ferroptosis, to mediate ferroptosis-associated renal fibrosis.

### Inhibition of GPX4 restores the severity of renal fibrosis in the UUO kidney of Smad3 KO mice *in vivo* and in Smad3 KO MEFs *in vitro*

**Figure 6:**
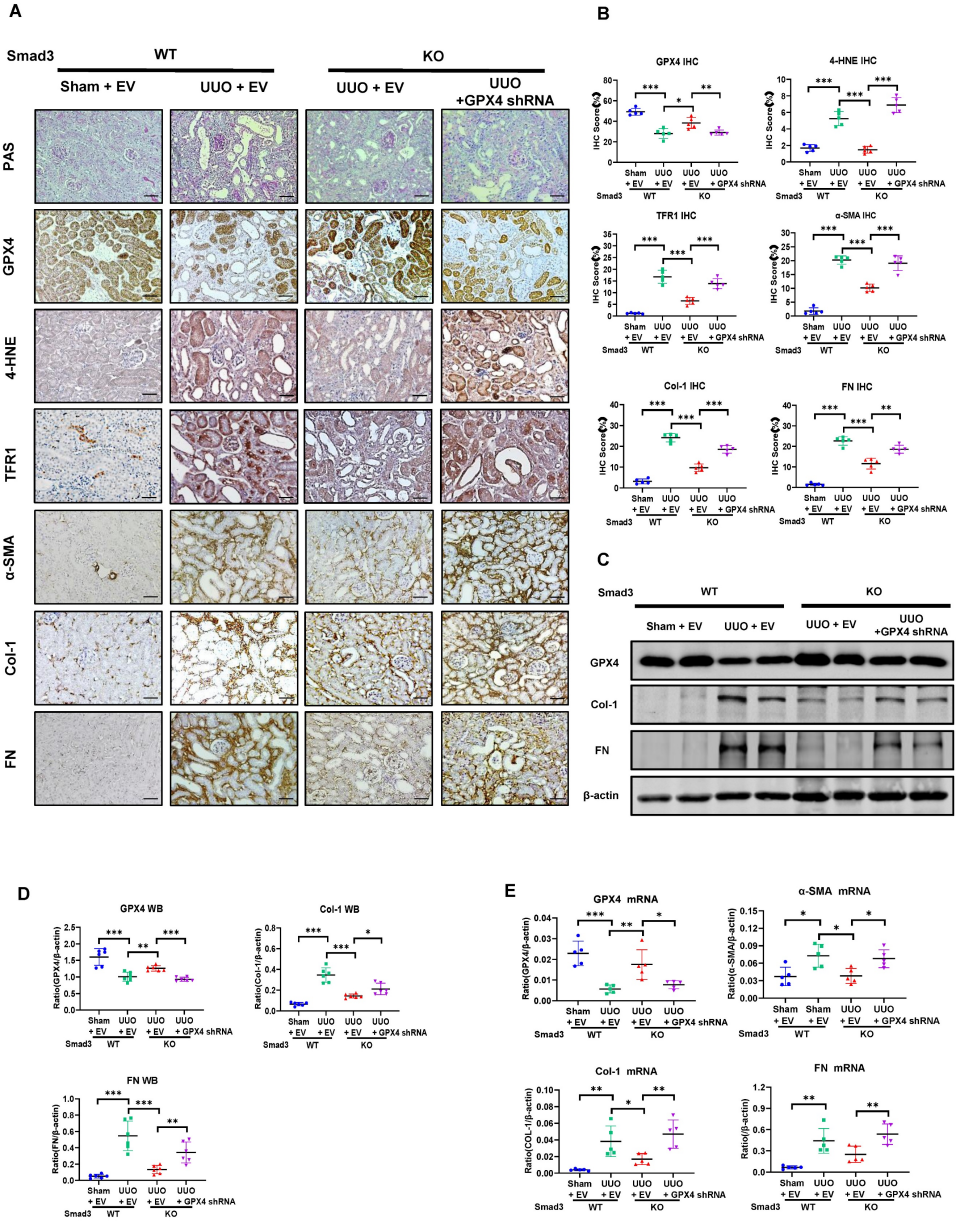
** Kidney-specifically silencing GPX4 restores the severity of ferroptosis and renal fibrosis in the UUO kidney of Smad3 KO mice. (A, B)** Representative photomicrographs and quantitative analysis for histology (PAS) and expression of GPX4, 4-HNE, TFR1 and fibrotic markers including α-SMA, Col-1, FN from Smad3 WT/KO mice at 7 days after UUO. **(C, D)** Western blot and quantitative analysis of renal GPX4, Col-1, and FN protein expression. **(E)** Real-time PCR for renal GPX4, α-SMA, Col-1, and FN mRNA expression. Data are presented as mean ± SD for groups of 6 mice. *P < 0.05, **P < 0.01, ***P < 0.001. Scale bar, 50 μm.

**Figure 7:**
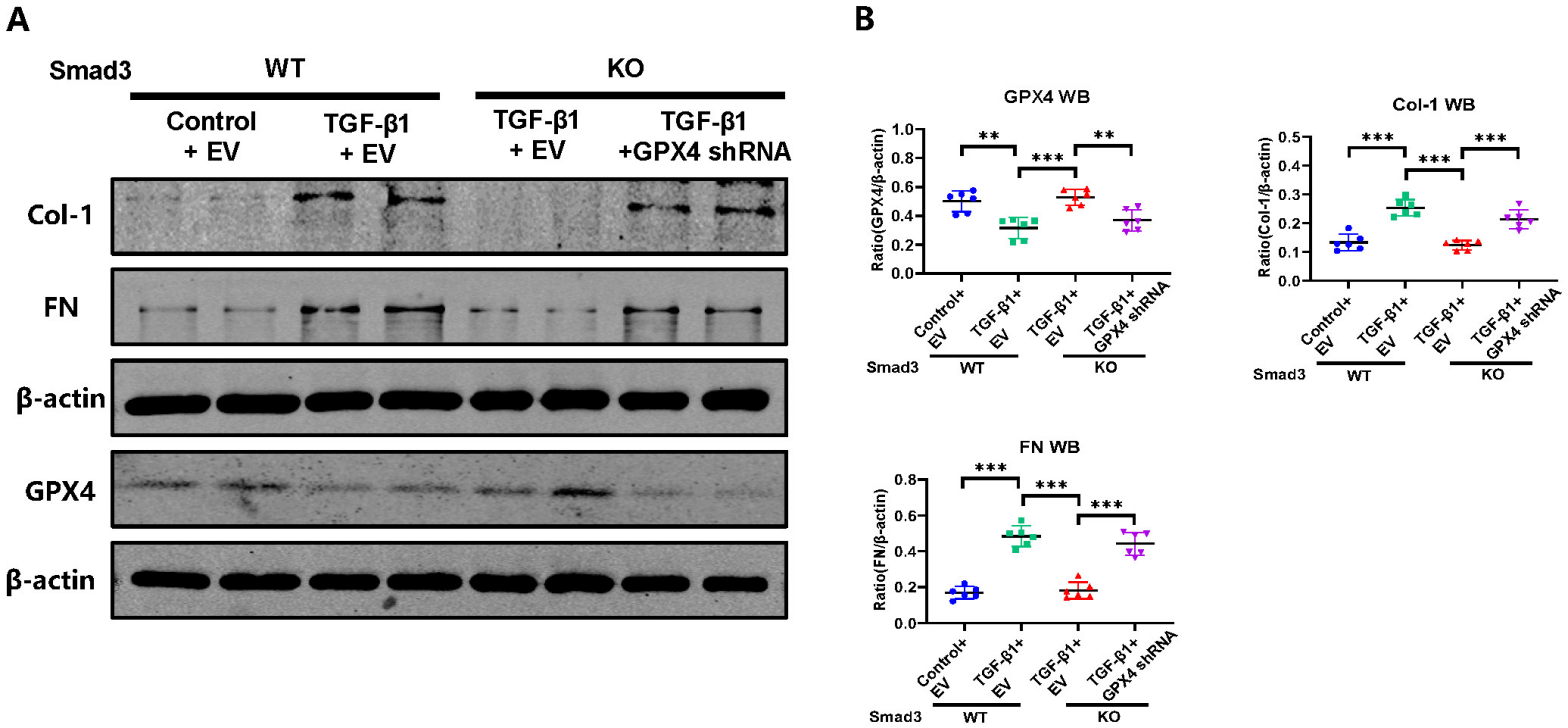
** Silencing GPX4 restores TGF-β1-induced fibrosis in Smad3 KO MEFs *in vitro*.** Western blot analysis shows that silencing GPX4 restores TGF-β1 (5ng/ml)-induced fibrosis such as Col-1, FN in Smad3 KO MEFs to the comparable levels of Smad3 WT MEFs. (A) Wester blots; (B) quantitative analysis. Data are presented as mean ± SD for 3 independent experiments. **P < 0.01, ***P < 0.001.

To further explore the role of GPX4 in Smad3-mediated renal fibrosis by kidney-specifically knocking down GPX4 in Smad3 KO mice using the well-established ultrasound-microbubble-mediated gene transfer technique. Consistent with the observations shown in **Figure 5** and **Figure S4B,** mice null for Smad3 were largely protected against the development of severe ferroptosis and progressive renal fibrosis. In contrast, specifically silencing renal GPX4 in Smad3 KO mice restored the severity of ferroptosis and progressive renal fibrosis to similar levels as seen in Smad3 WT UUO mice including a loss of GPX4 while increasing expression of 4-HNE, TFR1, α-SMA, Col-1, and FN (**Figure 6** and **Figure S5A**). This was further demonstrated *in vitro* in Smad3 KO MEFs and SIS3-treated HK-2 cell in which genetic deletion or pharmacological inhibition of Smad3 markedly suppressed TGF-β1-induced loss of GPX4 and expression of 4-HNE, TFR1, α-SMA, Col-1, and FN, which was reversed by silencing GPX4 (**Figure 7 and Figures S3** and** S5B**)**.** All these *in vivo* and *in vitro* findings reveal a protective role for GPX4 in Smad3-mediated renal fibrosis.

## Discussion

It is well recognized that TGF-β/Smad3 signaling is a key pathway leading to renal fibrosis and mediates fibrosis by directly binding to the collagen genes or miRNAs/lncRNAs that specifically regulate renal fibrosis.^6^, ^14^-^17^, ^48^-^50^ Increasing evidence also reveals that Smad3 is a key regulator for the cell death in AKI and CKD by triggering activation of cell death pathways such as apoptosis, necroptosis, and autophagy.^18^-^21^ Recently, ferroptosis has been reported to be associated with renal fibrosis in CKD patients and mice via the GPX4-dependent mechanism.^26^, ^31^-^36^ Ferroptosis has been demonstrated to contribute to the pathogenesis of renal fibrosis by promoting inflammatory responses 40 and facilitating epithelial-mesenchymal transition (EMT).^38^, ^51^ However, mechanisms that regulate GPX4-dependent ferroptosis during renal fibrosis remain unknown. Consistent with the previous observation, 26, 31-34 in this study, we also detected progressive renal fibrosis in patients with IgAN and DKD and in UUO mice was associated with decreased GPX4 while increasing ferroptosis as demonstrated by high expression levels of 4-HNE and TFR1. Importantly, we also demonstrated that loss of GPX4 in the fibrotic kidney was associated with overactivation of TGF-β/Smad3, suggesting a Smad3-GPX4-dependent ferroptosis axis in the pathogenesis of renal fibrosis.

The most significant finding in this study is the discovery of a new mechanism by which Smad3 mediates renal fibrosis via GPX4-dependent ferroptosis. This was confirmed by the findings that there is a binding site for Smad3 in the promoter region of GPX4 gene. Thus, Smad3 could bind to GPX4 directly to repress its transcription. Although the promoter regions are responsible for enhancing gene transcription by binding to activating transcription factors or coactivators, repressive transcription factors can also occur by interacting with the promoter regions to modulate gene expression. In muscle stem cells, the binding of inhibitory factors, such as NF-kB and Zinc finger E-box-binding homeobox 1 (ZEB-1), can repress the expression of genes associated with muscle differentiation.^52^ It is possible that Smad3 could bind to the promoter region of GPX4 gene to repress its transcription. This was demonstrated by the findings from dual-luciferase assay in which overexpression of Smad3 largely suppressed *GPX4* transcription, which was abrogated when the Smad3 binding site was mutated (**Figure 4B**). Functionally, we also found that genetic deletion or pharmacological inhibition of Smad3 was able to protect mice or cells from UUO or TGF-β1-induced repression of GPX4 and upregulation of 4-HNE and TFR1, thereby inhibiting the ferroptosis-associated fibrosis *in vivo* and *in vitro*. Thus, GPX4 is negatively regulated by TGF-β via a Smad3-dependent mechanism. It is highly possible that targeting Smad3 with Smad3 inhibitors such as SIS3 inhibits renal fibrosis as well as acute kidney injury via a mechanism associated with GPX4-dependent ferroptosis as seen in this study and other previous reports. 18-25 This is also well supported by recent studies that treatment with Tectorigenin and Formononetin ameliorates ferroptosis-associated renal fibrosis by inhibiting Smad3 signaling. 53, 54

Importantly, we also identified that GPX4 is a Smad3 target gene and functions to protect Smad3-mediated renal fibrosis. This was demonstrated by the findings that silencing GPX4 restored high levels of 4-HNE, TFR1 and the severity of renal fibrosis in the UUO kidney of Smad3 KO mice and TGF-β1-treated Smad3 KO MEFs and HK-2 cells treated with SIS3. All these findings demonstrated that GPX4 is a downstream target gene of Smad3 and is protective in Smad3-mediated renal fibrosis.

In conclusion, the activation of TGF-β/Smad3 signaling is responsible for loss of GPX4 and the development of ferroptosis-associated fibrosis in the fibrotic kidney of patients and mice with CKD. As a downstream target gene of Smad3, GPX4 is negatively regulated by TGF-β via a Smad3-dependent mechanism but exerts a protective role in Smad3-mediated renal fibrosis. Thus, it is possible that targeting the Smad3/GPX4-ferroptosis regulatory pathway may be a promising therapeutic strategy for treatment of renal fibrosis.

## Supplementary Material

Supplementary figures.
