# Supplementary material for: Smad3 Mediates Renal Fibrosis via GPX4-Dependent Ferroptosis
Source: Int J Biol Sci. 2025 Sep 22;21(13):5922–35. doi: 10.7150/ijbs.114075 (PMC12509913; doi:10.7150/ijbs.114075)
Supplement: Supplementary file 1 — Supplementary figures. [file ijbsv21p5922s1.pdf]

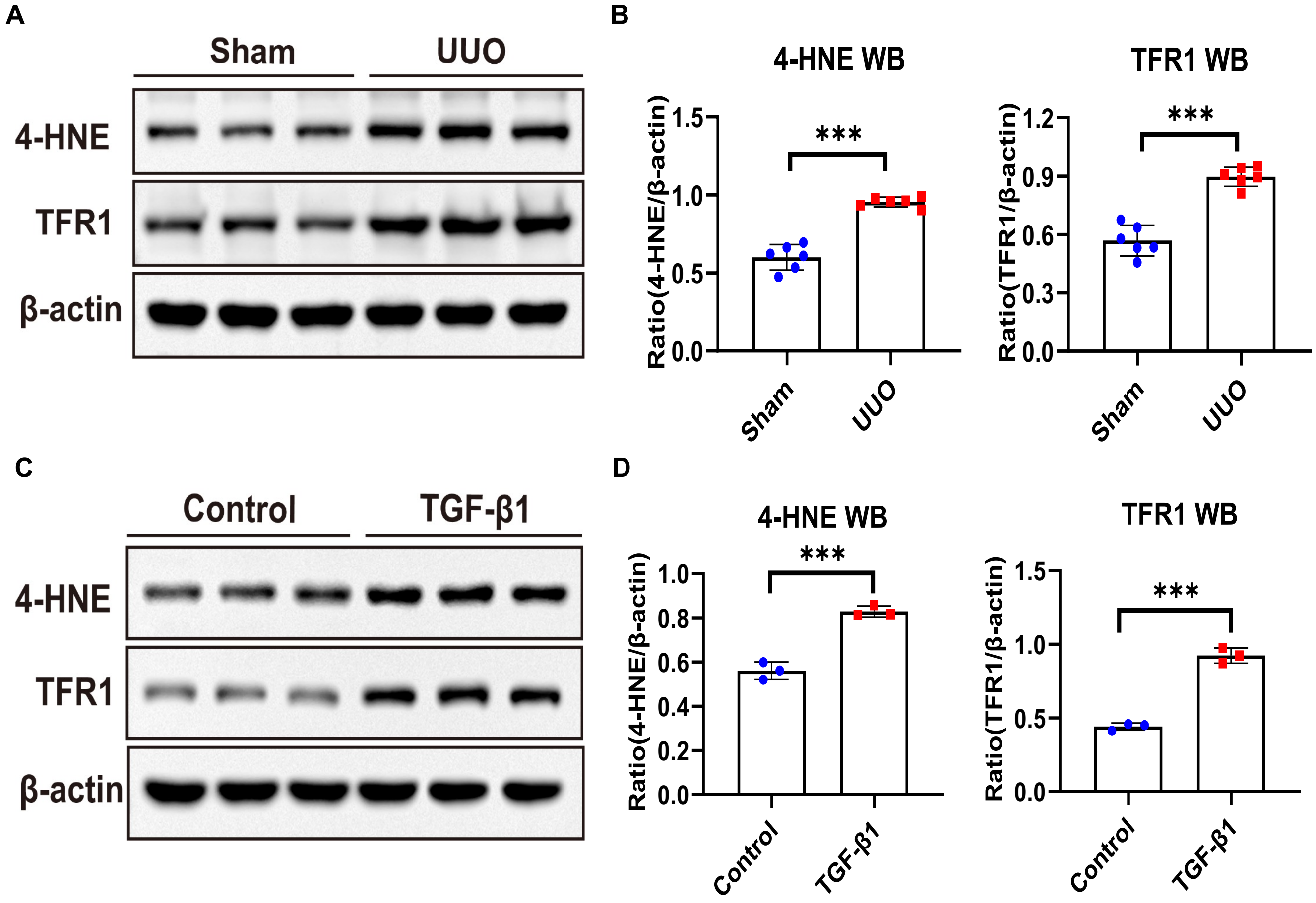

Figure S1. Expression of two ferroptosis biomarkers 4-HNE and TFR1 in the mouse UUO kidney and in TGF-β1-treated MEFs in vitro. (A, B) Western blot and quantitative analysis of 4-HNE and TFR1 expression in the renal tissues of sham-control and UUO mice. **(C, D)** Western blot and quantitative analysis of 4-HNE and TFR1 expression in TGF-β1(5ng/ml)-treated MEFs. Data are presented as mean ± SD for groups of 6 mice (A,B) or three independent experiments (C,D). ∗∗∗*P* < 0.001.

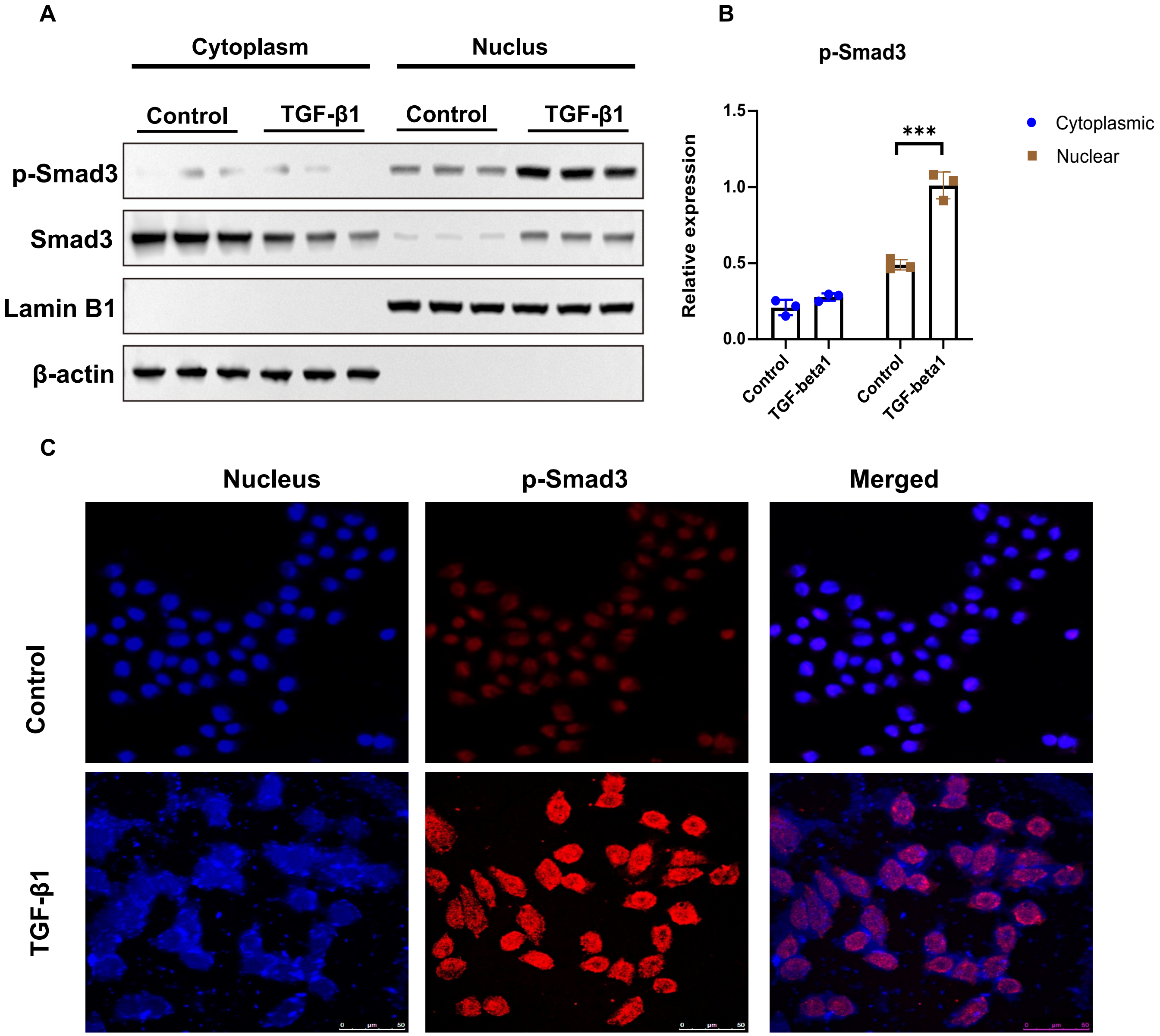

Figure S2. TGF-β1 promotes p-Smad3 from the cytoplasm to the nucleus in HK-2 cells. (A, B) Western blot analysis of p-Smad3 translocation from cytoplasm and nucleus in response to TGF-β1 (5 ng/ml) in HK-2 cells. **(C)** Immunofluorescence shows TGF-β1 (5ng/ml)-induced p-Smad3 nuclear translocation in HK-2 cells. Data are presented as mean ± SD for 3 independent experiments. ***$P < 0.001$.

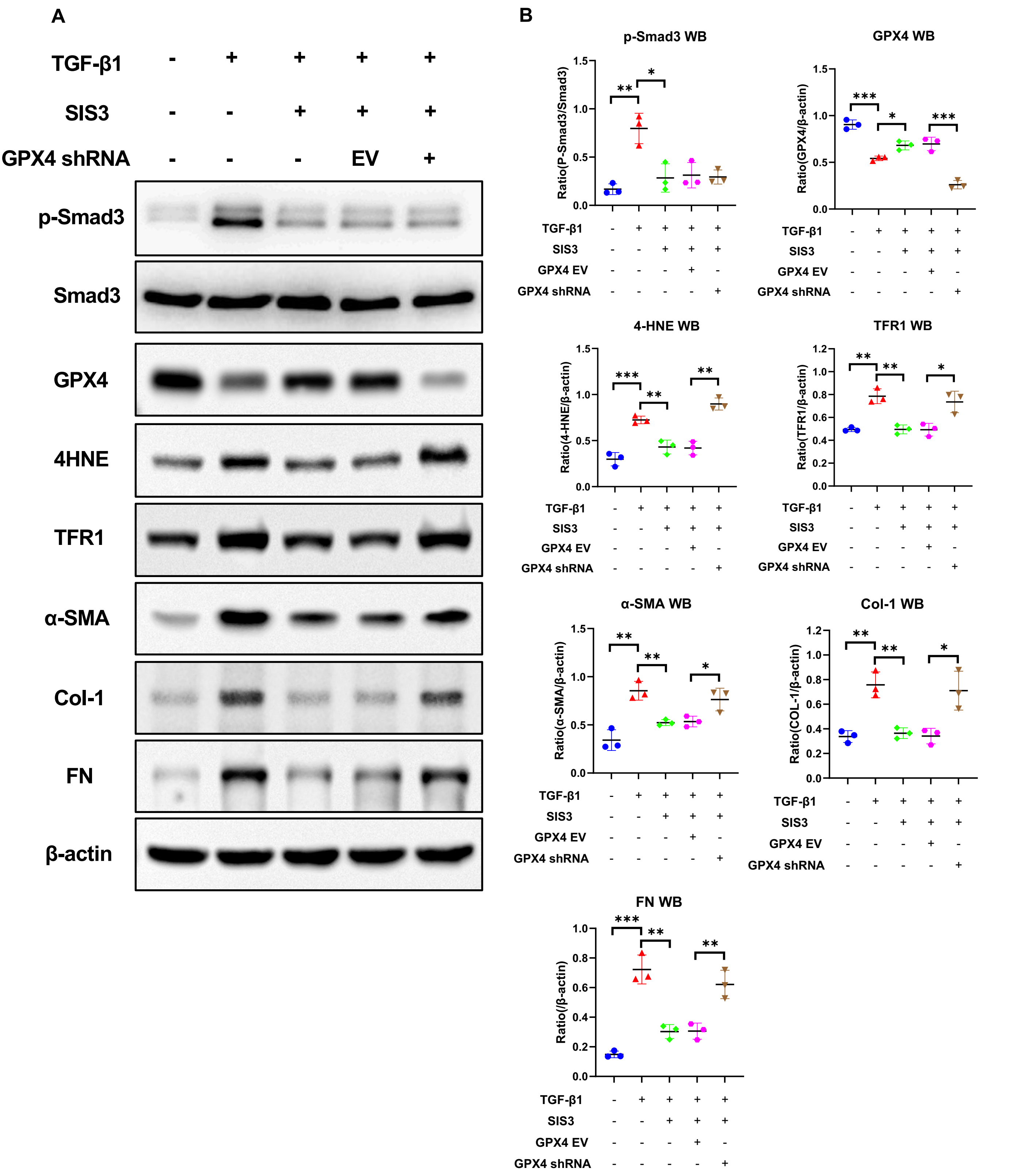

Figure S3. Inhibition of Smad3 with SIS3 suppresses TGF-β1-induced ferroptosis and fibrosis response in HK-2 cells, which was reversed by silencing GPX4. (A) Western blotting; (**B**) Quantitative analysis. Results show that treatment with SIS3 blocks TGF-β1 (5 ng/mL)-induced loss of GPX4 and upregulation of two ferroptosis markers (4-HEN and TFR1), and thus inhibits fibrosis by suppressing expression of α-SMA, Col-1, and FN, all these changes are reversed by overexpressing GPX4 shRNA. EV: Empty vector. Data are presented as mean ± SD for three independent experiments; $*P < 0.05$, $**P < 0.01$, $***P < 0.001$.

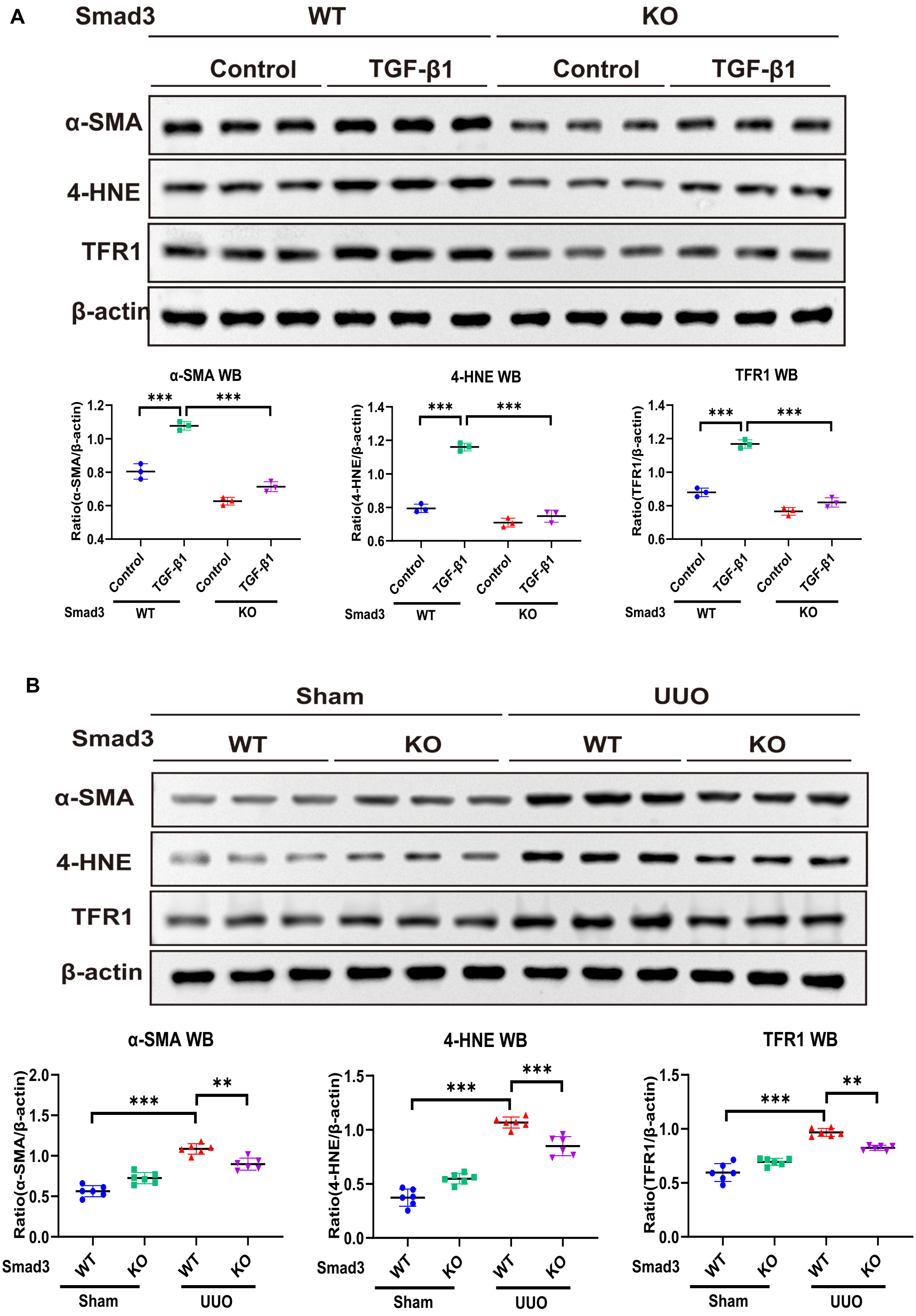

Figure S4. Genetic deletion of Smad3 protects against UUO- or TGF-β1-induced ferroptosis and upregulation of α-SMA in vivo and in vitro. (A) Western blot analysis shows that deletion of Smad3 protects against TGF-β1(5 ng/mL)-induced upregulation of two ferroptosis markers (4-HNE and TFR1) and α-SMA in Smad3 KO MEFs. **(B)** Western blot analysis detects that mice null for Smad3 are protected from UUO-induced ferroptosis by suppressing expression of 4-HNE and TFR1 and α-SMA protein accumulation. Data are presented as mean ± SD for 3 independent experiments (A) or groups of 6 mice (B). $*P < 0.05$, $**P < 0.01$, $***P < 0.001$.

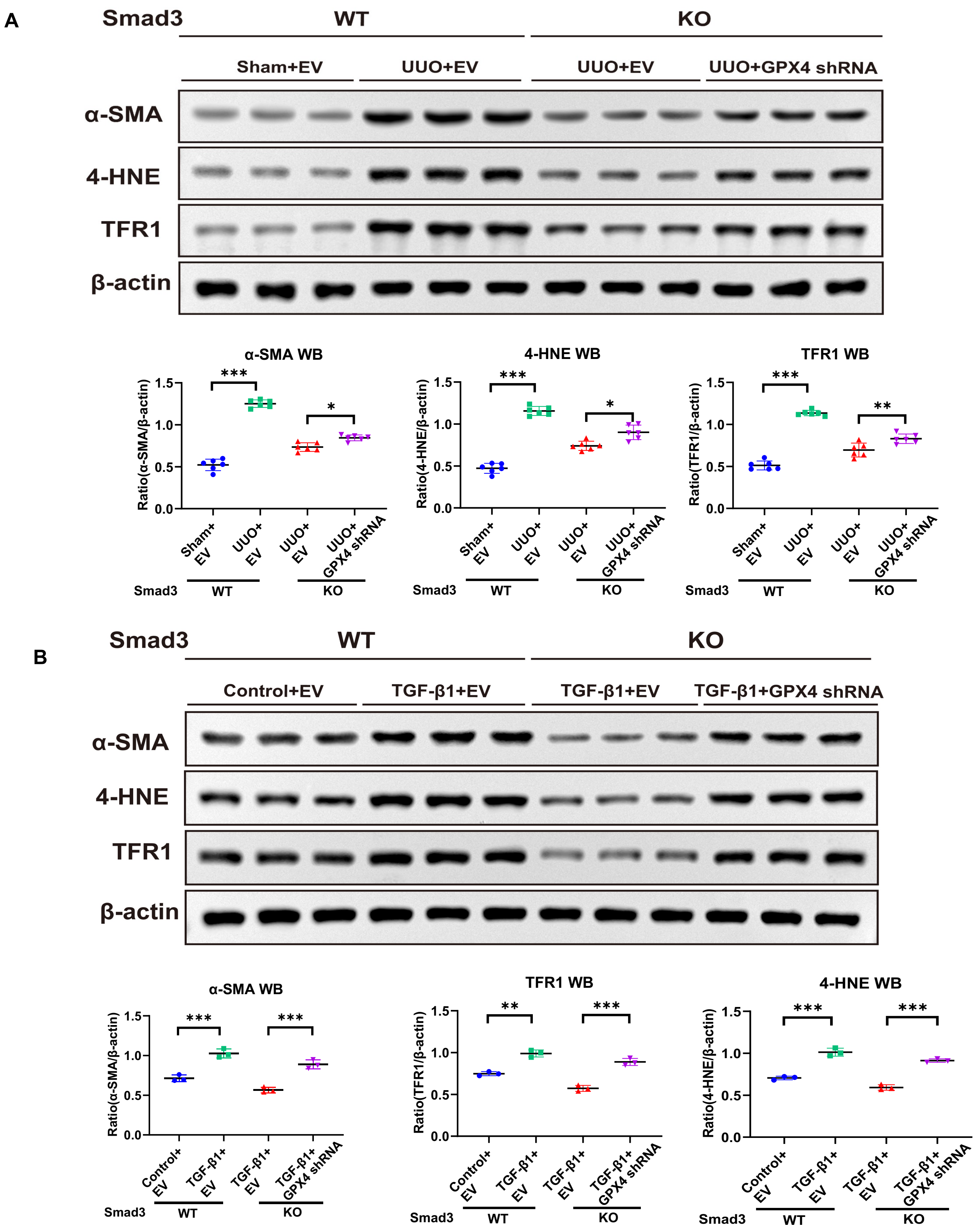

Figure S5. Silencing GPX4 restores the severity of renal fibrosis in the UUO kidney of Smad3 KO mice and in Smad3 KO MEFs in vitro. (A) Western blot and quantitative analysis of renal α-SMA, 4-HNE and TFR1 protein expression in a mouse model of UUO induced in Smad3 WT or KO mice. **(B)** Western blot and quantitative analysis of TGF-β1 (5ng/ml)-induced α-SMA, 4-HNE and TFR1 protein expression in Smad3 WT or KO MEFs. Data are presented as mean ± SD for groups of 6 mice (A) or 3 independent experiments (B); $*P < 0.05$, $**P < 0.01$, $***P < 0.001$.
